# Supplementary material for: The genome sequence of a tachinid fly, Cistogaster globosa (Fabricius, 1775)
Source: Wellcome Open Res. 2023 Oct 13;8:462. [Version 1] doi: 10.12688/wellcomeopenres.19924.1 (PMC11292184; doi:10.12688/wellcomeopenres.19924.1)
Supplement: Peer review history

# Reviewer report: Reviewer response for version 1

**DOI:** 10.21956/wellcomeopenres.22063.r90494

**Published:** 2024-08-14

**Authors:** Ruiqi Li

**Affiliations:** 1 University of Colorado Boulder, Boulder, Colorado, USA

Falk et al. have presented a high-quality genome of the tachinid fly, *Cistogaster globosa *(Fabricius, 1775). I have a few minor comments:

 1. Could the authors double-check the species distribution? A quick check on the GBIF occurrence map shows records primarily in Europe.

 2. Please use the full name for "RDB" when it first appears in the text.

 3. I suggest the authors expand on the significance of the genome, such as revealing molecular mechanisms of parasitism or co-evolution. A few sentences should suffice. You may refer to some previous genome notes if they are helpful:

1. Li R, Li J, Lopez JV *et al.*( 2023 ^1^ )
2. Li R, Li J, Lopez JV *et al.*(2024 ^2^ )
3. Li R, Li J, Lopez JV *et al.* (2024 ^3^ )

Are sufficient details of methods and materials provided to allow replication by others?

Yes

Is the rationale for creating the dataset(s) clearly described?

Yes

Are the datasets clearly presented in a useable and accessible format?

Yes

Are the protocols appropriate and is the work technically sound?

Yes

Reviewer Expertise:

Genomics

I confirm that I have read this submission and believe that I have an appropriate level of expertise to confirm that it is of an acceptable scientific standard.

## References

[1] : The genome sequence of the giant clam, Tridacna crocea (Lamarck, 1819). *Wellcome Open Research* .2023;8: 10.12688/wellcomeopenres.20580.1 10.12688/wellcomeopenres.20580.1

[2] : The genome sequence of the giant clam, Tridacna gigas (Linnaeus, 1758). *Wellcome Open Res* .2024;9: 10.12688/wellcomeopenres.21136.1 145 10.12688/wellcomeopenres.21136.1 38800516 PMC11116938

[3] : The genome sequence of the smooth giant clam, Tridacna derasa Röding, 1798. *Wellcome Open Research* .2024;9: 10.12688/wellcomeopenres.22618.1 10.12688/wellcomeopenres.22618.1 PMC1111693838800516

# Reviewer report: Reviewer response for version 1

**DOI:** 10.21956/wellcomeopenres.22063.r90491

**Published:** 2024-08-12

**Authors:** Charles Plessy

**Affiliations:** 1 Okinawa Institute of Science and Technology, Okinawa, Japan

This data note follows the standard structure of the Darwin Tree of Life Consortium and is easy to follow.  The primary assembly ticks most of the checkboxes of quality except perhaps for the number of scaffolds, which is above a thousand. I am concerned by the alternate haplotype assembly (GCA_937654215.1), which is longer of ~300 Mb. This does not make sense to me as 1) these haplotypes were sequenced from a single individual and 2) the primary assembly contains both the X and the Y chromosomes.  Therefore if there were any significant difference in length, I would expect the alternate assembly to be shorter.  Can the authors explain the difference or double-check the alternate haplotype assembly for duplications or contaminations?

Are sufficient details of methods and materials provided to allow replication by others?

Yes

Is the rationale for creating the dataset(s) clearly described?

Yes

Are the datasets clearly presented in a useable and accessible format?

Yes

Are the protocols appropriate and is the work technically sound?

Yes

Reviewer Expertise:

Genomics

I confirm that I have read this submission and believe that I have an appropriate level of expertise to confirm that it is of an acceptable scientific standard, however I have significant reservations, as outlined above.

# Reviewer report: Reviewer response for version 1

**DOI:** 10.21956/wellcomeopenres.22063.r90498

**Published:** 2024-07-31

**Authors:** Arun Arumugaperumal

**Affiliations:** 1 Department of Biotechnology, Rajalakshmi Engineering College, Chennai, Tamilnadu, India

Several tachinid fly genomes were sequenced and reported as part of Darwin Tree of Life project. This one is *Cistogaster globosa* (Fabricius, 1775). A google scholar search with keywords " *Cistogaster globosa* genome" did not yield any other report other than this. So this should be the first whole genome sequence of the fly. The data article reports an assembly of length 837.8 Mb covering 7 chromosomes. Also a mitogenome of 16.97 kb was reported. The authors annotated a total of 29,591 genes in the genome.

 The photograph of the insect presented was not focussed well. The tube was focused and the insect was out of focus. 

 Long read technology was used for the sequencing experiment. The authors have used PacBio Sequel II platform with 25X coverage. The contig N50 value was 4.1 Mb which is very good for annotation. The authors have used Ensembl to automatically annotate their genome data. The genome assembly completeness was checked using BUSCO analysis. The analysis results show that the assembly at hand is about 98.7 % complete. Taken all together, the data reported herewith is of huge importance when it comes to designing molecular biology experiments on this organism. 

 Since several of the tachinid fly genomes are available, the authors could have made a comparison or could have estimated the pan-genome.

Are sufficient details of methods and materials provided to allow replication by others?

Yes

Is the rationale for creating the dataset(s) clearly described?

Yes

Are the datasets clearly presented in a useable and accessible format?

Yes

Are the protocols appropriate and is the work technically sound?

Yes

Reviewer Expertise:

Bioinformatics,  Genomics

I confirm that I have read this submission and believe that I have an appropriate level of expertise to confirm that it is of an acceptable scientific standard.

# Reviewer report: Reviewer response for version 1

**DOI:** 10.21956/wellcomeopenres.22063.r68728

**Published:** 2023-10-20

**Authors:** Jaakko Pohjoismäki

**Affiliations:** 1 Department of Environmental and Biological Sciences, University of Eastern Finland, Joensuu, Finland

This genome note presents another nice addition to the growing list of reference genomes from the DToL. Unlike many other bug-killing flies (Phasiinae), Cistogaster globosa is relatively easy to identify, even from the rather small image in the figure 1. As the species is sexually dimorphic (females are almost entirely shining black), it is possible to confirm the sex of the specimen to be male, as stated. This notable sexual dimorphism might be actually noteworthy to mention in the background?

 I am surprised to read that the species has been having some endangered status. To my experience it is not very particular with its habitat demand as Aelia are all around where there's a bit of low growth, dryish grasslands, such as roadsides, suburban wastelands etc.

 I had a hard time in finding out what RDB 1 and 2 mean, this could have been made more clear. As googling was futile, I asked ChatGTP, who considered RBD 1 comparable to CR and RBD 2 to VU in the IUCN terminology. I hope she got it right.

 For readers outside of the UK, these details would be interesting to know.

 The genome report looks good. Waiting to get to browse through the genes online.

Are sufficient details of methods and materials provided to allow replication by others?

Yes

Is the rationale for creating the dataset(s) clearly described?

Yes

Are the datasets clearly presented in a useable and accessible format?

Yes

Are the protocols appropriate and is the work technically sound?

Yes

Reviewer Expertise:

Diptera, biodiversity, molecular biology

I confirm that I have read this submission and believe that I have an appropriate level of expertise to confirm that it is of an acceptable scientific standard.
